# Vaccinia Virus Infection in Monkeys, Brazilian Amazon

**DOI:** 10.3201/eid1606.091187

**Published:** 2010-06

**Authors:** Jônatas S. Abrahão, André T. Silva-Fernandes, Larissa S. Lima, Rafael K. Campos, Maria I.M.C. Guedes, Marcela M.G. Cota, Felipe L. Assis, Iara A. Borges, Milton F. Souza-Júnior, Zélia I.P. Lobato, Cláudio A. Bonjardim, Paulo C.P. Ferreira, Giliane S. Trindade, Erna G.

**Affiliations:** Universidade Federal de Minas Gerais, Belo Horizonte, Minas Gerais, Brazil

**Keywords:** Vaccinia virus, orthopoxvirus, viruses, monkeys, poxvirus, reservoirs, Brazil, dispatch

## Abstract

To detect orthopoxvirus in the Brazilian Amazon, we conducted a serosurvey of 344 wild animals. Neutralizing antibodies against orthopoxvirus were detected by plaque-reduction neutralizing tests in 84 serum samples. Amplicons from 6 monkey samples were sequenced. These amplicons identified vaccinia virus genetically similar to strains from bovine vaccinia outbreaks in Brazil.

In Brazil, several exanthematic vaccinia virus (VACV) outbreaks affecting dairy cattle and rural workers have been reported since 1999 (*1**,**2*). VACV, the prototype of the genus *Orthopoxvirus*, shows serologic cross-reactivity with other Orthopoxvirus species and was used during the smallpox eradication campaign (*3*). Bovine vaccinia causes economic losses and affects public health services in Brazil (*4*). VACV reservoirs and the role of wildlife in outbreaks remain unidentified. Although some data indicate that VACV strains circulate in rodents in forests in Brazil (*5**,**6*), there is no evidence of VACV infection in other wild mammals.

To detect orthopoxviruses in the Brazilian Amazon, we conducted a serosurvey of wild animals in this region. We detected antibodies against orthopoxviruses in 4 mammalian species. Using molecular methods, we confirmed exposure of monkeys to VACV. Although our findings are uncertain in the context of bovine vaccinia outbreaks, we provide new biologic and epidemiologic information about VACV.

## The Study

During February 2001–September 2002, we captured 344 wild mammals in an overflow area in a fauna-rescue program during construction of a hydroelectric plant in Lajeado and Ipueiras counties (9°44′58′′S, 48°21′23′′W) in Tocantins State, Brazil. During this program, 269 capuchin monkeys (*Cebus apella*), 27 black-howling monkeys (*Allouata caraya*), 12 coatis (*Nasua nasua*), 20 agoutis (*Dasyprocta* sp.), 2 opossums (*Didelphis albiventris*), 5 armadillos (*Euphractus sexcinctus*), 5 collared anteaters (*Tamandua tetradactila*), and 4 gray foxes (*Cerdocyon thous*) were captured.

All animals were captured in a sylvatic area and did not have contact with humans and dairy cattle. In field-screening laboratories, animals were sedated, serum samples were collected, and veterinary evaluations were made. Animals were then released in areas selected during environmental conservation programs. Until 2002, bovine vaccinia had been restricted to southeastern Brazil, >1,400 km from the study area (*7*).

Serum samples were inactivated by heating at 56°C for 30 min, and an orthopoxvirus plaque-reduction neutralizing test (PRNT) was performed. PRNT was used rather than ELISA because secondary antibodies required for an ELISA for all analyzed species were unavailable. Inactivated samples were diluted 1:20×–1:1,640× in minimal essential medium and tested in Vero cells by using the VACV-Western Reserve strain in the PRNT as described (*8*). Human samples positive for antibodies to orthopoxvirus obtained during bovine vaccinia outbreaks were used as positive controls (*9*); samples negative for these antibodies were used as negative controls (*10*). Serum titer was defined as the highest dilution that inhibited >50% of viral plaques compared with negative controls. Orthopoxvirus PRNT specificity (97.4%) and sensitivity (93.5%) were confirmed by using receiver-operating characteristic analysis, which compared results of PRNT, ELISA, and clinical symptoms during bovine vaccinia outbreaks (*9**,**10*).

PRNT showed a high prevalence of seropositive monkeys (Table). Of 269 *C*. *apella* samples, 68 (25.3%) had antibodies to orthopoxvirus. Of 27 *A*. *caraya* samples, 13 (48.1%) had antibodies to orthopoxvirus. Seropositivity was detected in 2 (16.6%) coatis and 1 (5.0%) agouti. Antibodies to orthopoxvirus were not detected in any other species tested. Of 344 animals studied, 84 (24.4%) had antibodies to orthopoxvirus (Table). In samples with high neutralizing antibody titers, 55.95% (47) had titers of 80–320. Only 5 (6.0%) PRNT-positive samples had titers <40 (Table).

**Table:** Seroprevalence of vaccinia virus in monkeys, Brazilian Amazon*

Species	No. serum samples tested	PRNT titer, no. positive					Total no. (%)	No. (%) PCR positive
		20	40	80	160	320		
*Cebus apella*	269	2	24	36	4	2	68 (25.3)	11 (4.1)
*Allouata caraya*	27	3	5	5	0	0	13 (48.1)	7 (25.9)
*Nasua nasua*	12	0	2	0	0	0	2 (16.6)	0
*Dasyprocta* sp.	20	0	1	0	0	0	1 (5.0)	0
*Didelphis albiventris*	2	0	0	0	0	0	0	0
*Euphractus sexcinctus*	5	0	0	0	0	0	0	0
*Tamandua tetradactila*	5	0	0	0	0	0	0	0
*Cerdocyon thous*	4	0	0	0	0	0	0	0
Total	344	5	32	41	4	2	84 (24.4)	18 (5.2)

*PRNT, plaque-reduction neutralization test.

Given the serologic cross-reactivity of orthopoxvirus (*3*), positive samples could indicate any of >9 virus species, although it is well established that VACV is endemic to Brazil, and infections with other orthopoxviruses are geographically restricted to other continents and have not been identified in Brazil. Therefore, we performed a molecular investigation to identify orthopoxviruses associated with orthopoxvirus sylvatic circulation. Serologic and molecular tests were performed in a blinded fashion and in triplicate. On the basis of previous studies that detected orthopoxvirus DNA in serum of infected hosts (*9**,**11**,**12*), a semi-nested PCR was used to amplify the highly conserved orthopoxvirus vaccinia growth factor (*vgf*) gene (J.S. Abrahão et al., unpub.data) from mammal serum samples. Human VACV DNA–positive and DNA–negative serum samples obtained during bovine vaccinia outbreaks (*9*) were used as positive and negative controls, respectively. Field and laboratory clinical samples were processed separately to avoid cross-contamination.

Eighteen of 344 serum samples were positive in PCR assays (11 from *C*. *apella* and 7 from *A*. *caraya*; all were PRNT positive). Six of the 18 *vgf* PCR-positive samples were chosen for sequencing and analysis of *vgf* (4 from *C*. *apella* and 2 from *A*. *caraya*). In addition, using primers described by Ropp et al. (*14*), we amplified the hemagglutinin (*ha*) gene from 2 samples (1 from *C*. *apella* and 1 from *A*. *caraya*; both were *vgf* positive). The *vgf* and *ha* PCR products were cloned into the pGEMT-easy vector (Promega, Madison, WI, USA). Three clones from distinct PCR amplicons of each sample were sequenced in both orientations by using M13 universal primers and the Mega-BACE-sequencer (GE Healthcare, Little Chalfont, UK).

Optimal alignment of the highly conserved *vgf* gene with ClustalW (www.ncbi.nlm.nih.gov/pmc/articles/PMC308517) and MEGA version 3.1 (www.megasoftware.net) showed 100% identity among all nucleotide and amino acid sequences for monkey serum (Appendix Figure, panel A). When compared with nucleotide sequences available in GenBank, *vgf* sequences were highly similar (98%–100% identity) to the homologous gene from other VACV strains and showed 100% identity. The *ha* sequences for *C*. *apella* and *A*. *caraya* showed a signature deletion (Appendix Figure, panel B) also present in sequences of other VACV isolates from Brazil. These *ha* sequences showed 99.6% identity at the nucleotide level and 99.7% identity at the amino acid level (736 nt of the *ha* gene were analyzed).

Phylogenetic trees of the *vgf* (Figure, panel A) or *ha* (Figure, panel B) genes were constructed by using the neighbor-joining method, 1,000 bootstrap replicates, and the Tamura 3-parameter model (MEGA version 3.1). These sylvatic VACV sequences clustered with several VACVs isolated during several bovine vaccinia outbreaks. The *vgf* and *ha* sequences from monkey samples were deposited in GenBank (accession nos. VACV-TO *vgf* GQ465372 and GQ465373 and *ha* GU322359 and GU322360). Orthopoxvirus DNA was not detected in any other species tested.

**Figure:**
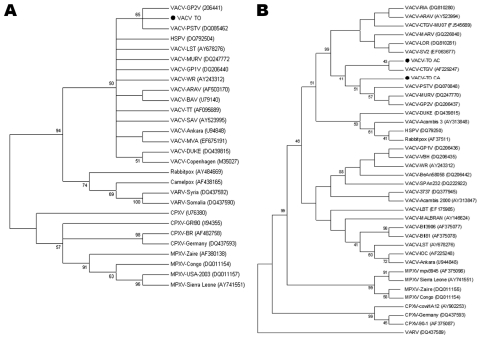
Consensus bootstrap phylogenetic trees based on nucleotide sequences of orthopoxvirus vaccinia growth factor (*vgf*) (A) and hemagglutinin (*ha*) (B) genes. Trees were constructed with *ha* or *vgf* sequences by using the neighbor-joining method with 1,000 bootstrap replicates and the Tamura 3-parameter model in MEGA version 3.1 software (www.megasoftware.net). Bootstrap values >40% are shown. Nucleotide sequences were obtained from GenBank. Black dots indicate vaccinia virus (VACV) obtained from *Cebus apella* (VACV-TO CA) and *Allouata caraya* (VACV AC). All *vgf* sequences obtained from monkey serum samples showed 100% and are represented as a unique sequence in the *vgf* tree (VACV TO). HSPV, horsepoxvirus; VARV, variola virus; CPXV, cowpoxvirus; MPXV, monkeypoxvirus.

## Conclusions

Although VACV strains have been isolated from rodents in forests in Brazil (*5**,**6*) (the nearest location, Belém, is 750 km from the study area), we detected VACV in wildlife in the Brazilian Amazon 3 years after reports of exanthematic outbreaks of bovine vaccinia and 40 years after isolation of VACV from forests. Our data provide evidence of high prevalence of orthopoxviruses among capuchin and black-howling monkeys in the Brazilian Amazon. The relationship between infected monkeys and emergence of VACV in rural regions of Brazil is unknown. However, transmission of VACV in northeastern Brazil has been reported, and outbreaks have been reported in Mato Grosso, Pernambuco (www.amep.org.br/pox.doc), Maranhão (E.G. Kroon et al., unpub. data), and Tocantins (*13*), which are in or adjacent to the Brazilian Amazon. Some of these viruses may be related to those isolated in this study because some VACV isolates have the same signature deletion in the *ha* gene as VACV-TO.

Anthropogenic disturbance of the Amazon ecosystem and increases in agricultural and livestock areas increase contact between wildlife and rural populations (*15*). However, the effect of VACV in environments in Brazil that contain wild animals has not been studied. Clinical data for pox lesions in animals tested were not well documented by veterinarians in the study area. Ecologic and public health studies should be designed to evaluate risks for infection with VACV during wildlife conservation efforts and determine whether surveillance systems can predict bovine vaccinia outbreaks by monitoring VACV infection in monkeys and other wild animals.

## Supplementary Material

Appendix Figure Amino acid sequences of vaccinia virus (VACV) samples and comparison with homologous genes sequences from several orthopoxviruses, Brazil. A) Alignment of vaccinia growth factor gene sequences from 6 monkey serum samples showing 100% identity (horizontal box). VACV-TO_CA, sequence from *Cebus apella*; VACV-TO_AC, sequence from *Allouata caraya*; HPXV, horsepoxvirus; CPXV, cowpoxvirus; MPXV, monkeypoxvirus; VARV, variola virus; ECMV, ectromelia virus. B) Alignment of orthopoxvirus hemagglutinin gene amino acid sequences showing the deletion signature region (vertical box) in VACV-TO isolates and several VACV strains isolated during bovine vaccinia outbreaks. Arrow indicates polymorphism site in the hemagglutinin amino acid sequences between VACV-TO_CA and VACV-TO_AC. Alignments were made by using ClustalW (www.ncbi.nlm.nih.gov/pmc/articles/PMC308517) and MEGA version 3.1 software (www.megasoftware.net). HSPV, horsepoxvirus.

## References

[1] de Souza Trindade G, da Fonseca FG, Marques JT, Nogueira ML, Mendes LC, Borges AS, Araçatuba virus: a vaccinialike virus associated with infection in humans and cattle. Emerg Infect Dis. 2003;9:155–60. 12603984 10.3201/eid0902.020244 PMC2901946

[2] Leite JA, Drumond BP, Trindade GS, Lobato ZI, da Fonseca FG, dos Santos JR, Passatempo virus, a vaccinia virus strain, Brazil. Emerg Infect Dis. 2005;11:1935–8. 16485483 10.3201/eid1112.050773 PMC3367646

[3] Damon IK. Poxviruses. In: Knipe DM, Howley PM, Griffin DE, Lamb RA, Martin MA, Roizman B, et al., editors. Fields Virology. Vol II. 5th ed. Philadelphia: Lippincott Williams and Wilkins; 2007. p. 2947–75.

[4] Lobato ZI, Trindade GS, Frois MC, Ribeiro EB, Dias GR, Teixeira BM, Outbreak of exantemal disease caused by vaccinia virus in human and cattle in Zona da Mata Region, Minas Gerais. Arquivo Brasiliero Medicina Veterinária Zootecnia. 2005;57:423–9.

[5] de Lopes S, Lacerda JP, Fonseca IE, Castro DP, Forattini OP, Rabello EX. Cotia virus: a new agent isolated from sentinel mice in São Paulo, Brazil. Am J Trop Med Hyg. 1965;14:156–7. 14248989 10.4269/ajtmh.1965.14.156

[6] Fonseca FG, Lanna MC, Campos MA, Kitajima EW, Peres JN, Golgher RR, Morphological and molecular characterization of the poxvirus BeAn 58058. Arch Virol. 1998;143:1171–86. 10.1007/s007050050365 9687874

[7] Damaso CR, Esposito JJ, Condit RC, Moussatche N. An emergent poxvirus from humans and cattle in Rio de Janeiro State: Cantagalo virus may derive from Brazilian smallpox vaccine. Virology. 2000;277:439–49. 10.1006/viro.2000.0603 11080491

[8] Office International des Epizooties. West Nile fever in the United States of America in horses. In: Manual of standard techniques. Paris: The Office; 1999. p. 150–1.

[9] Silva-Fernandes AT, Travassos CE, Ferreira JM, Abrahão JS, Rocha ES, Viana-Ferreira F, Natural human infections with vaccinia virus during bovine vaccinia outbreaks. J Clin Virol. 2009;44:308–13. 10.1016/j.jcv.2009.01.007 19243990

[10] Madureira M. Study of the bovine vaccine outbreaks in Minas Gerais State [PhD dissertation]. Belo Horizonte (Brazil): Escola de Veterinária, Universidade Federal de Minas Gerais; 2009.

[11] Cohen JI, Hohman P, Preuss JC, Li L, Fischer SH, Fedorko DP. Detection of vaccinia virus DNA, but not infectious virus, in the blood of smallpox vaccine recipients. Vaccine. 2007;25:4571–4. 10.1016/j.vaccine.2007.03.044 17493714 PMC2082009

[12] Savona MR, Dela Cruz WP, Jones MS, Thornton JA, Xia D, Hadfield TL, Detection of vaccinia DNA in the blood following smallpox vaccination. JAMA. 2006;295:1898–900. 10.1001/jama.295.16.1898 16639047

[13] Medaglia ML, Pessoa LC, Sales ER, Freitas TR, Damaso CR. Spread of Cantagalo virus to northern Brazil. Emerg Infect Dis. 2009;15:1142–3. 10.3201/eid1507.081702 19624947 PMC2744230

[14] Ropp SL, Jin Q, Knight JC, Massung RF, Esposito JJ. PCR strategy for identification and differentiation of small pox and other orthopoxviruses. J Clin Microbiol. 1995;33:2069–76. 7559950 10.1128/jcm.33.8.2069-2076.1995 PMC228337

[15] Soares-Filho BS, Nepstad DC, Curran LM, Cerqueira GC, Garcia RA, Ramos CA, Modelling conservation in the Amazon basin. Nature. 2006;440:520–3. 10.1038/nature04389 16554817

