# Supplementary figures and images for: Vaccinia Virus Infection in Monkeys, Brazilian Amazon
Source: Emerg Infect Dis. 2010 Jun;16(6):976–9. doi: 10.3201/eid1606.091187 (PMC3086250; doi:10.3201/eid1606.091187)

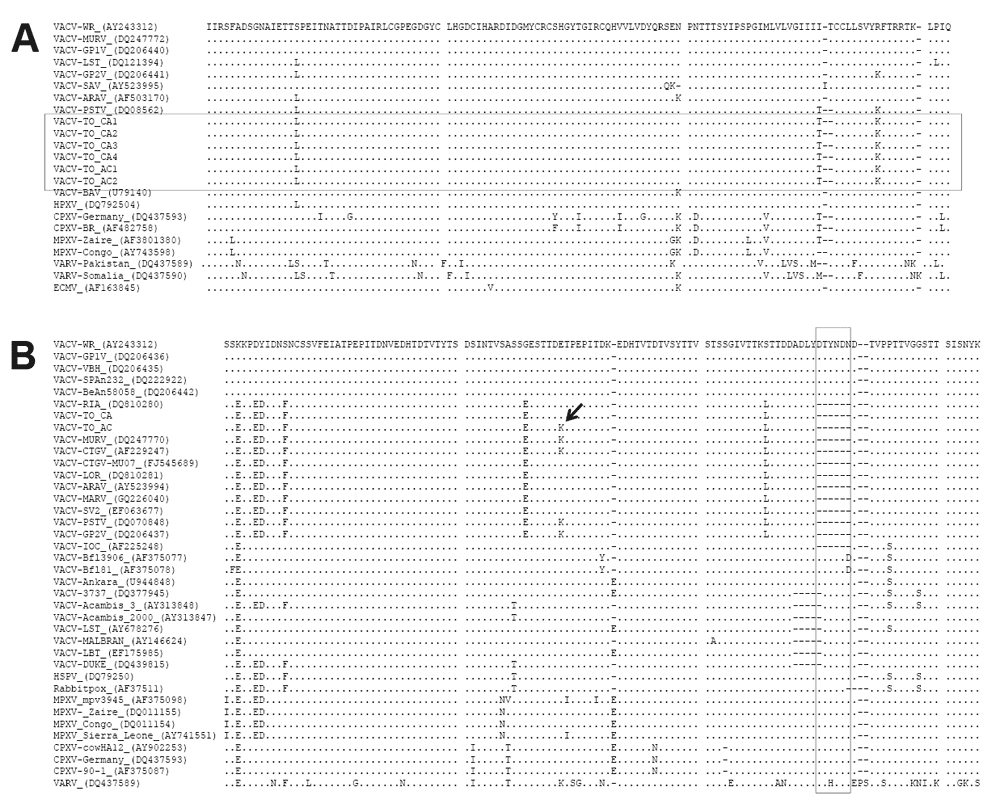

Supplement: Appendix Figure — Amino acid sequences of vaccinia virus (VACV) samples and comparison with homologous genes sequences from several orthopoxviruses, Brazil. A) Alignment of vaccinia growth factor gene sequences from 6 monkey serum samples showing 100% identity (horizontal box). VACV-TO_CA, sequence from Cebus apella; VACV-TO_AC, sequence from Allouata caraya; HPXV, horsepoxvirus; CPXV, cowpoxvirus; MPXV, monkeypoxvirus; VARV, variola virus; ECMV, ectromelia virus. B) Alignment of orthopoxvirus hemagglutinin gene amino acid sequences showing the deletion signature region (vertical box) in VACV-TO isolates and several VACV strains isolated during bovine vaccinia outbreaks. Arrow indicates polymorphism site in the hemagglutinin amino acid sequences between VACV-TO_CA and VACV-TO_AC. Alignments were made by using ClustalW (www.ncbi.nlm.nih.gov/pmc/articles/PMC308517) and MEGA version 3.1 software (www.megasoftware.net). HSPV, horsepoxvirus. [file 09-1187-appF-s1.gif]
